# Maternal mineral nutrition during early pregnancy and neonatal growth: findings from a China birth cohort study

**DOI:** 10.3389/fnut.2026.1733235

**Published:** 2026-02-18

**Authors:** Doudou Zhao, Yanfang Song, Liang Li, Nan Li, Yu Zhang, Yiting Li, Kangxin Wang, Yang Mi, Lei Shang, Pengfei Qu

**Affiliations:** 1 Translational Medicine Center, Northwest Women’s and Children’s Hospital, Xi’an, China; 2 Department of Health Statistics, Ministry of Education Key Lab of Hazard Assessment and Control in Special Operational Environment, School of Public Health, Fourth Military Medical University, Xi’an, China; 3 Department of Epidemiology and Biostatistics, School of Public Health, Xi’an Jiaotong University Health Science Center, Xi’an, China; 4 Department of Obstetrics, Northwest Women’s and Children’s Hospital, Xi’an, China; 5 Central Laboratory, Beijing Obstetrics and Gynecology Hospital, Capital Medical University, Chaoyang, Beijing, China

**Keywords:** birth length, calcium, cohort study, iron, maternal mineral nutrition, non-linear relationship, SGA

## Abstract

**Objectives:**

Neonatal growth holds great significance for lifelong health, but the effects of maternal mineral nutrition during pregnancy on neonatal growth remain unclear. This study aimed to investigate the associations of the maternal mineral nutrition including copper (Cu), zinc (Zn), calcium (Ca), magnesium (Mg), and iron (Fe) during early pregnancy with neonatal growth.

**Methods:**

A prospective cohort study was conducted in Xi’an, China, with a total of 5,629 mother-infant dyads. The study examined the non-linear relationship and threshold effects between minerals and neonatal growth using smoothed plots and two-piecewise regression models.

**Results:**

Every one-unit elevation in log-transformed Ca concentration was associated with a 97% (RR: 0.03, 95% CI: 0.01–0.80) lower risk of small-for-gestational-age (SGA). Maternal Fe concentration was associated with birth length of male infants in an inverted U-shaped curve. When Fe concentration was less than 7.24 mmol/L, a one-unit elevation in log-transformed Fe concentration was associated with a 3.23-cm higher birth length (95% CI: 0.13–6.32) in male infants. However, this relationship was not found in female infants.

**Conclusion:**

Adequate maternal Ca and Fe in early pregnancy may benefit newborn growth and development, but the gender differences should also be considered. Vigilant monitoring and prompt remediation of maternal mineral deficiencies during early pregnancy are essential for optimizing neonatal health outcomes.

## 1 Introduction

The neonatal birth parameters not only facilitate the assessment of intrauterine growth, but also assist in identifying individuals who may be at risk for adverse developmental outcomes or diseases in childhood and adulthood. Both excessive and extremely low birth weight, in particular, have been demonstrated to have an impact on a variety of health outcomes, such as impaired lung function, asthma (1), and type 1 diabetes (2) in children, poorer reproductive health (3), reduced cardiorespiratory fitness (4), and compromised cardiovascular health (5) in adults. During the initial two years of life, birth length serves as the most potent indicator for growth status and stunting (6). Greater birth length also serves as a potential risk factor for obesity (7) and hypertension (8) among children and adolescents. The early recognition of modifiable maternal biomarkers may be extremely important for improving neonatal growth outcomes. Nevertheless, identifying risk markers for women during early pregnancy continues to pose challenges.

Previous studies have demonstrated that genetic factors, nutritional status, maternal characteristics, and environmental pollution are all related to neonatal growth (9, 10). Mineral nutrition has been recognized as having specific functions in various aspects such as cellular metabolism, providing antioxidant and anti-inflammatory protection, influencing enzyme activity, and regulating gene expression (11). Examining the relationships between maternal mineral nutrition and neonatal growth is of crucial importance due to the continuously growing focus on maternal mineral nutrition assessment during pregnancy (12). Early pregnancy, which is a crucial stage for fetal growth, is especially susceptible to the influences of mineral nutrition (13). While most epidemiological research has been centered around heavy metals (14, 15), only a small number of studies have indicated that various mineral nutrients such as copper (Cu), zinc (Zn), calcium (Ca), magnesium (Mg), and iron (Fe) can affect the development of the fetus and health of mothers (16–18). In the early stages of pregnancy, a higher concentration of Zn has been linked to an increased head circumference in female infants (19). On the other hand, reduced concentrations of Ca significantly increase birth length, brachial circumference, and the risk of low birth weight (20). However, conflicting findings suggest that the concentrations of Cu, Zn, and Fe in pregnant women during early pregnancy are not associated with large for gestational age infants (21). Moreover, some studies have even reported contradictory findings as maternal blood Zn level was inversely associated with neonatal birth weight (22, 23).

This study aims to explore the associations between maternal mineral nutrients including Cu, Zn, Ca, Mg, and Fe during early pregnancy and neonatal growth among the Chinese population. In addition, a smoothed plot and a two-piecewise regression model were employed to specifically analyze the nonlinear association and threshold effect between maternal Fe concentration and birth length. Understanding the associations between mineral nutrition and neonatal growth can inform targeted interventions for mothers in the first trimester to improve birth outcomes.

## 2 Materials and methods

### 2.1 Study design and population

A prospective birth cohort study (24) was conducted at Northwest Women’s and Children’s Hospital, a tertiary hospital in Xi’an, Shaanxi province, China. From July 2018 to December 2023, pregnant women who had their first prenatal care visit before 14 gestational weeks were invited to participate in the birth cohort and voluntarily sign the informed consent. The inclusion criteria for the cohort included: Chinese nationality, gestational age between 6 and 13^+6^ weeks and planned to attend routine prenatal visits and deliver at the same hospital. The exclusion criterion was the voluntary withdrawal of women from the cohort at any time. The study was approved by the Human Research Ethics Committee of Beijing Obstetrics and Gynecology Hospital (2018-KY-003-02) and Northwest Women’s and Children’s Hospital (2018018).

**Figure 1:**
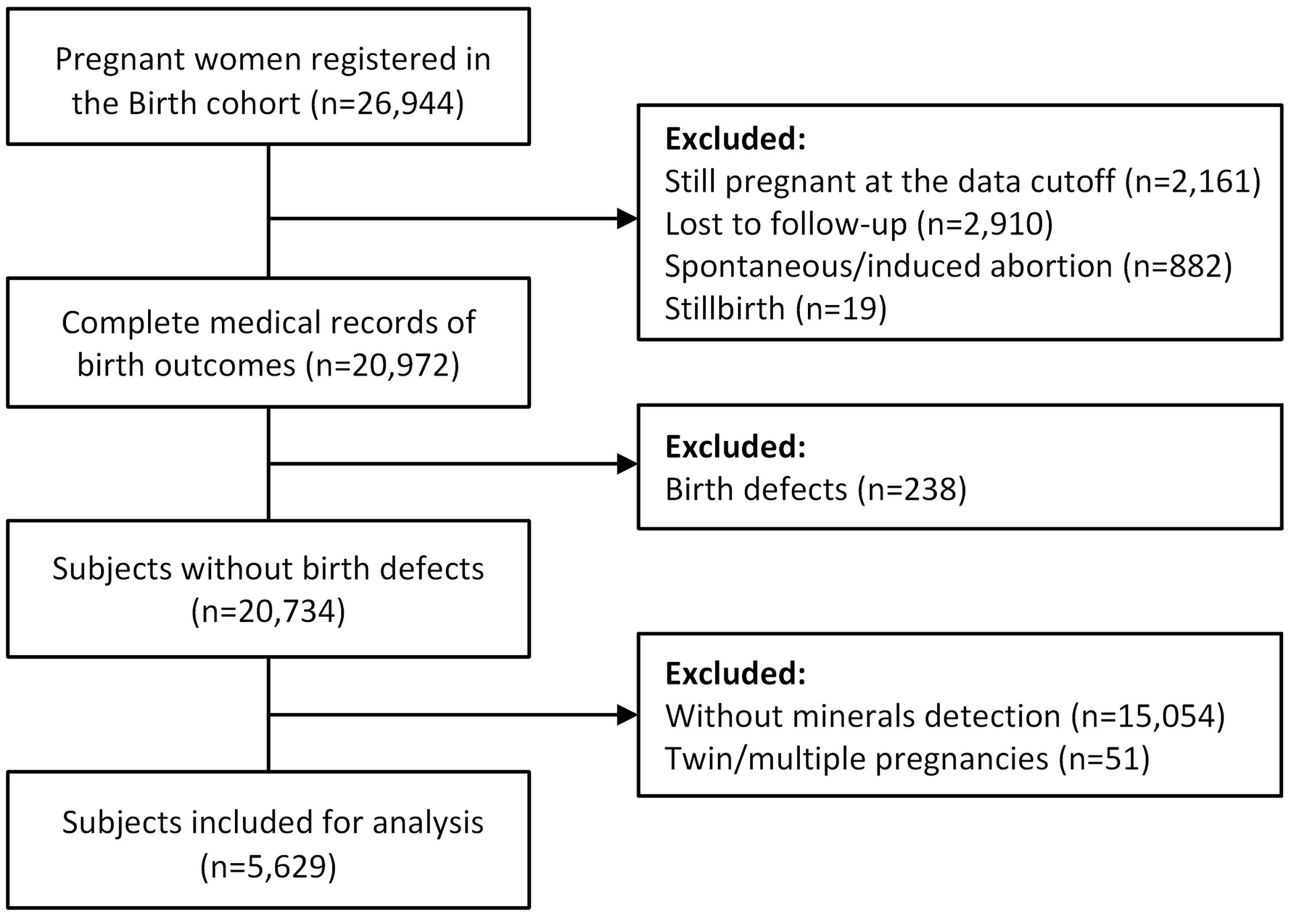
Flowchart of participants.

In this study, participants who were still pregnant at the data cutoff (*n* = 2,161), lost to follow-up (*n* = 2,910), experienced spontaneous or induced abortion (*n* = 882), had stillbirth (*n* = 19), had birth defects (*n* = 238), had no mineral detection data (*n* = 15,054), and had twin or multiple pregnancies (*n* = 51) were excluded. Finally, 5,629 subjects were included in the analysis (Figure 1). Women included in the final analysis showed no significant differences in maternal baseline characteristics when compared with those who were excluded (Supplementary Table S1).

### 2.2 Socio-demographic variables and lifestyle

At the time of enrollment, baseline characteristics of mothers during early pregnancy were obtained through self-reporting on the online enterprise data center (EDC) cloud platform. The body mass index (BMI) was calculated according to the Chinese BMI criteria by using pre-pregnancy weight (kg) and height (cm) data, and women were then categorized into three BMI groups: underweight (BMI < 18.5 kg/m^2^), normal weight (18.5 ≤ BMI < 24.00 kg/m^2^), overweight and obesity (BMI ≥ 24 kg/m^2^). The average annual household income was categorized as being in the poverty range (less than 50,000 Chinese yuan [CNY]), the medium range (50,000 to 200,000 CNY), and the high range (more than 200,000 CNY). History of adverse pregnancy was defined as cases where mothers had experienced abortion, stillbirth, or birth defect in a previous pregnancy. Maternal smoking or passive smoking was defined as mothers smoking at least 1 cigarette or passively inhaling smoke for more than 15 min at least 1 day per week during early pregnancy.

### 2.3 Neonatal growth measurement

The main outcome of interest for this study was neonatal growth, defined as birth weight, birth length, large for gestational age (LGA), and small for gestational age (SGA) infants. The postpartum birth weight and length were measured by an electronic height and weight examination device and subsequently recorded through the inpatient record system of the hospital. Based on the INTERGROWTH-21st standards (25), LGA was defined as a birth weight above the 90th percentile for the same gestational age and sex, and SGA was defined as a birth weight below the 10th percentile for the same gestational age and sex.

### 2.4 Determinations of minerals

The maternal fasting whole blood Cu, Zn, Ca, Mg, and Fe concentrations were quantified prior to 14 weeks of gestation using the atomic absorption spectroscopy method (AAS, Beijing, China), and then recorded using the hospital’s outpatient laboratory system. The 2 mL blood samples were obtained and promptly delivered to the laboratory center within 1 hour. The analytical performance of the method was validated in accordance with the quality management system of the accredited clinical laboratory. Based on calibration curves, the limits of detection (LOD) for Cu, Zn, Ca, Mg, and Fe were 0.32 μmol/L, 0.77 μmol/L, 2.50 × 10^−2^ mmol/L, 4.11 × 10^−5^ mmol/L, and 8.95 × 10^−4^ mmol/L, respectively. The measurements of the five minerals were above the LOD in all the study samples. Accuracy, defined as the closeness of agreement between measured values and certified reference values, was maintained within a deviation of ±10.0% as verified through ongoing analysis of internal quality control materials and participation in external proficiency testing schemes. Precision included both intra-assay repeatability and inter-assay reproducibility. The former was verified to meet laboratory standard operating procedure criteria, and the latter assessed under varying conditions was monitored continuously, with the coefficient of variation (CV) sustained at ≤5% for all elements across the study period based on long-term internal quality control data.

### 2.5 Statistical analysis

Categorical variables were summarized as counts and proportions and analyzed using Chi-squared tests. After the Kolmogorov–Smirnov test, the normal distribution of continuous variables was represented as mean and standard deviation. The non-normal distribution of continuous variables was represented as median and interquartile range (IQR). Due to the skewed distribution, the concentrations of minerals were subjected to natural log-transformation prior to regression analysis. We explored the association between maternal mineral nutrition and neonatal growth through the generalized linear models. For binary outcomes (SGA and LGA), we employed log-binomial regression with a log link function to estimate risk ratios (RRs) and 95% confidence intervals (CIs). For continuous outcomes (birth weight and birth length), we used linear regression with an identity link function to estimate *β* coefficients and 95% CIs. These analyses were performed in three different models: Model 1 without adjustments, Model 2 adjusted for the other four minerals, and Model 3 adjusted for all baseline characteristics and the other four minerals. The baseline covariates included maternal age, ethnicity, education, occupation, BMI before pregnancy, household income, first pregnancy, history of adverse pregnancy, mode of conception, smoking or passive smoke exposure, folic acid and multidimensional nutrients supplements, and neonatal gender. We primarily interpreted the findings from the fully adjusted Model 3, as it accounts for potential confounding from both other minerals and baseline covariates, thereby offering the most robust estimate of the independent associations. To further explore the non-linear relationship between maternal nutrition and neonatal growth, we used a smoothed plot and a two-piecewise regression model to examine the threshold effect of mineral concentration on birth outcomes using a smoothing function. The threshold level was determined using trial and error, including the selection of turning points along a predefined interval before choosing the turning point that gave the maximum model likelihood. The likelihood ratio test was used to compare the difference between Model 1 and Model 2 (two-piecewise regression models). In addition, we applied restricted cubic spline (RCS) models with four knots at the 5th, 35th, 65th, and 95th percentiles to assess nonlinearity between minerals and birth outcomes. Statistical analyses were completed using R software (version 4.2.3) and Empower (R) (www.empowerstats.com, X&Y Solutions, Inc. Boston, MA). Statistical significance was indicated by two-tailed *p* < 0.05.

## 3 Results

### 3.1 Characteristics of study population

A total of 5,629 mothers and their singleton infants were included. As displayed in Table 1, greater proportions of subjects were observed in mothers less than 35 years old (91.54%), Han ethnicity (98.19%), with junior or regular college educational level (72.52%), working regularly (78.52%), normal BMI (68.09%), medium average annual household income (65.29%), spontaneous conception (93.34%), without a history of adverse pregnancy (75.09%), non-smokers or not exposed to passive smoke (84.06%), and having folic acid supplements during early pregnancy (96.78%).

**Table 1:** Maternal baseline characteristics and neonatal birth outcomes (*N* = 5,629).

Characteristics	Mean ± SD or *n* (%)
Maternal characteristics	
Age, years	
<35	5,153 (91.54)
≥35	476 (8.46)
Ethnicity	
Han	5,527 (98.19)
Other	102 (1.81)
Education	
Senior high school or lower	703 (12.49)
Junior or regular college	4,082 (72.52)
Graduate or above	844 (14.99)
Occupation	
Working regularly	4,420 (78.52)
Workers/peasants/migrant workers	76 (1.35)
Unemployed	1,133 (20.13)
BMI before pregnancy, kg/m^2^	
Underweight (<18.5)	856 (15.21)
Normal (18.5 ~ 23.9)	3,833 (68.09)
Overweight and obesity (≥24.0)	940 (16.70)
Household income, CNY	
Poverty (<50,000)	685 (12.17)
Medium (50,000 ~ 200,000)	3,675 (65.29)
Rich (>200,000)	1,269 (22.54)
First pregnancy	
No	2,547 (45.25)
Yes	3,082 (54.75)
History of adverse pregnancy	
No	4,227 (75.09)
Yes	1,402 (24.91)
Mode of conception	
Spontaneous conception	5,254 (93.34)
Artificial fertilization	34 (0.60)
*In vitro* fertilization	341 (6.06)
Smoke or passive smoke	
No	4,732 (84.06)
Yes	897 (15.94)
Folic acid supplement	
No	181 (3.22)
Yes	5,448 (96.78)
Multidimensional nutrients supplement	
No	2,643 (46.95)
Yes	2,986 (53.05)
Birth outcomes	
Gestational age at delivery, week	39.34 ± 1.41
Birth weight, g	3313.48 ± 435.30
Birth length, cm	50.09 ± 1.59
Gender, male	2,904 (51.59)
SGA	259 (4.60)
LGA	573 (10.18)

The average neonatal gestational age, birth weight and birth length at delivery were 39.34 ± 1.41 weeks, 3313.48 ± 435.30 g, and 50.09 ± 1.59 cm, respectively. Among 5,629 infants, the number of infants with SGA and LGA was 259 (4.60%) and 573 (10.18%), respectively.

### 3.2 Mineral nutrition status during early pregnancy

Maternal mineral concentrations were detected before 14 weeks of gestation, and the average gestational age at the time of detection was 12.47 ± 1.85 weeks. As shown in Table 2, the median values of Cu, Zn, Ca, Mg, and Fe concentrations were 16.76 μmol/L, 84.57 μmol/L, 1.58 mmol/L, 1.38 mmol/L, and 7.68 mmol/L, respectively.

**Table 2:** Maternal mineral nutrition status during early pregnancy (*N* = 5,629).

Minerals	Median (IQR)
Cu, μmol/L	16.76 (13.37–20.34)
Zn, μmol/L	84.57 (76.57–92.97)
Ca, mmol/L	1.58 (1.48–1.72)
Mg, mmol/L	1.38 (1.32–1.49)
Fe, mmol/L	7.68 (7.23–8.20)

### 3.3 Mineral nutrition and neonatal growth

The Spearman correlation coefficients presented in Supplementary Figure S1 indicated that there were no strong correlations among the five minerals (|*r*| < 0.7), supporting their joint inclusion in the subsequent multivariate model. Table 3 showed the relationships between maternal mineral nutrition during early pregnancy and neonatal growth. Overall, the concentrations of most minerals showed no consistent associations with neonatal growth across the three models. Statistically significant associations were observed only for specific minerals in the adjusted models. In the unadjusted Model 1, no statistically significant associations were observed between maternal mineral concentrations in early pregnancy and neonatal growth outcomes (all *p* > 0.05). In Model 2, a one-unit elevation in log-transformed Mg concentration was associated with a 1.62-cm smaller birth length (95% CI: −3.11–−0.14), and the risk of LGA increased statistically significantly (RR: 7.60, 95% CI: 1.41–10.11). In Model 3, with every one-unit elevation in log-transformed Ca concentration, the risk of SGA significantly decreased (RR: 0.03, 95% CI: 0.01–0.80). The smoothed curve in Supplementary Figure S2 visually confirmed a linear dose–response relationship between Ca concentration and SGA risk.

**Table 3:** Associations between maternal mineral nutrition during early pregnancy and neonatal growth.

Outcomes	Model 1^a^	*p*	Model 2^b^	*p*	Model 3^c^	*p*
	*β*/RR (95% CI)		*β*/RR (95% CI)		*β*/RR (95% CI)	
Birth weight/kg^d^						
Cu	−11.49 (−95.42–72.44)	0.789	−27.31 (−116.96–62.33)	0.550	18.41 (−56.15–92.96)	0.629
Zn	86.37 (−97.38–270.11)	0.357	−21.58 (−272.48–229.31)	0.866	−140.29 (−347.67–67.08)	0.185
Ca	105.34 (−158.61–369.29)	0.434	92.49 (−209.89–394.87)	0.549	51.64 (−199.81–303.09)	0.687
Mg	81.78 (−210.14–373.70)	0.583	−178.44 (−582.08–225.21)	0.386	−44.56 (−378.74–289.61)	0.794
Fe	225.30 (−20.08–470.69)	0.072	330.74 (−36.69–698.17)	0.078	159.04 (−144.73–462.81)	0.305
Birth length/cm^d^						
Cu	−0.09 (−0.40–0.22)	0.588	−0.11 (−0.44–0.22)	0.502	0.11 (−0.18–0.41)	0.453
Zn	0.12 (−0.56–0.80)	0.738	0.05 (−0.88–0.98)	0.913	−0.12 (−0.95–0.71)	0.781
Ca	0.36 (−0.61–1.34)	0.467	0.66 (−0.46–1.78)	0.246	0.17 (−0.84–1.18)	0.744
Mg	−0.62 (−1.70–0.46)	0.258	−1.62 (−3.11–-0.14)	0.033	−1.13 (−2.47–0.20)	0.096
Fe	0.26 (−0.65–1.16)	0.580	0.98 (−0.37–2.34)	0.156	0.53 (−0.68–1.75)	0.390
SGA						
Cu	0.51 (0.22–1.23)	0.090	0.51 (0.20–1.28)	0.102	0.60 (0.22–1.55)	0.177
Zn	1.17 (0.13–7.17)	0.449	2.92 (0.14–16.83)	0.250	3.72 (0.17–36.40)	0.212
Ca	0.14 (0.01–2.74)	0.117	0.08 (0.01–2.02)	0.090	0.03 (0.01–0.80)	0.034
Mg	2.99 (0.16–16.65)	0.232	12.94 (0.69–22.95)	0.075	24.11 (0.85–117.63)	0.054
Fe	0.81 (0.07–8.15)	0.464	0.15 (0.01–5.95)	0.192	0.18 (0.01–8.27)	0.213
LGA						
Cu	1.29 (0.72–2.32)	0.205	1.41 (0.79–2.59)	0.152	1.11 (0.58–2.24)	0.385
Zn	0.73 (0.18–2.34)	0.334	0.60 (0.07–3.06)	0.311	0.41 (0.04–2.89)	0.216
Ca	0.39 (0.05–2.05)	0.180	0.18 (0.02–1.53)	0.086	0.49 (0.04–4.32)	0.274
Mg	3.27 (0.51–8.07)	0.122	7.60 (1.41–10.11)	0.021	9.20 (0.78–27.10)	0.070
Fe	0.87 (0.13–3.69)	0.441	0.31 (0.02–3.24)	0.228	0.32 (0.02–4.38)	0.255

a Model 1: not adjusted.

b Model 2: adjusted for other four metals.

c Model 3: adjusted for all baseline covariates (maternal age, ethnicity, education, occupation, BMI before pregnancy, household income, first pregnancy, history of adverse pregnancy, mode of conception, smoking or passive smoke exposure, folic acid and multidimensional nutrients supplements, and neonatal gender), and other four metals.

d Adjusted for all baseline covariates, other four metals, and gestational age in Model 3.

### 3.4 Non-linear relationship between Fe and birth length

**Figure 2:**
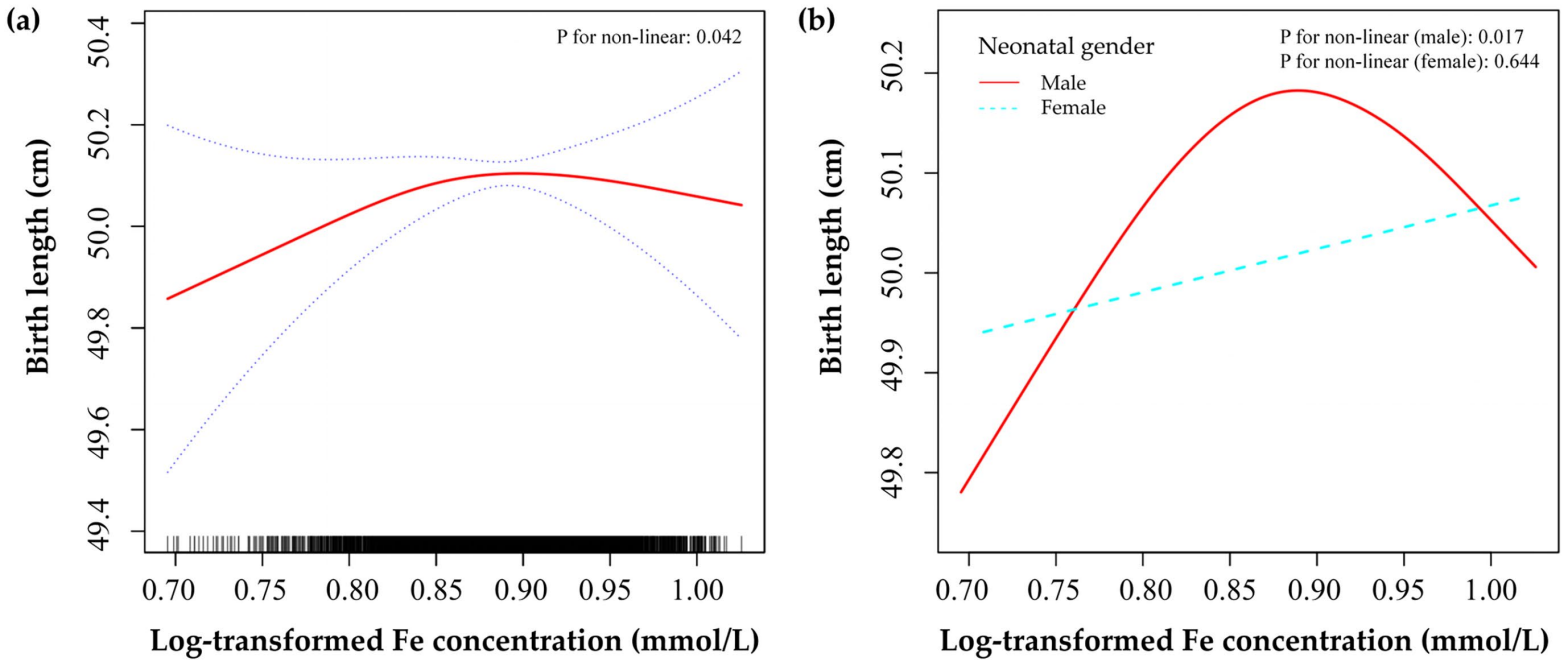
Smoothed plots of maternal Fe concentration and neonatal birth length in all subjects **(a)** and stratified by infant sex **(b)**. The red solid line and the blue dashed lines in panel **(a)** represent the estimated birth length and 95% CI, respectively. Adjusted for all baseline covariates, gestational age, and other four metals.

Smoothing plots in Supplementary Figure S2 and threshold effect analysis in Supplementary Table S2 demonstrated the relationships between minerals and neonatal growth, with a non-linear pattern between Fe and birth length observed. Figure 2 showed that maternal Fe concentration was associated with birth length in an inverted U-shaped curve in all subjects and male infants. When Fe concentration was less than 7.24 mmol/L (log Fe = 0.86), a one-unit elevation in log-transformed Fe concentration was associated with a 2.42-cm (95% CI: 0.35–4.49) and 3.23-cm (95% CI: 0.13–6.32) higher birth length in all subjects and male infants, respectively (Table 4). However, after this point, the birth length did not further respond to higher Fe concentrations. In addition, no statistically significant linear or non-linear associations were found in female infants (Supplementary Table S3).

**Table 4:** Threshold effects of maternal Fe concentration on birth length in all subjects and male infants.

Birth length	All subjects	Male infants
	Adjusted *β* (95% CI)^a^, *p*	Adjusted *β* (95% CI) ^a^, *p*
Model 1		
One line slope	0.53 (−0.68–1.75), 0.390	0.35 (−1.48–2.18), 0.709
Model 2		
Turning point (K)	0.86	0.86
<0.86^b^ slope 1	2.42 (0.35–4.49), 0.022	3.23 (0.13–6.32), 0.041
≥0.86^b^ slope 2	−0.93 (−2.71–0.85), 0.307	−1.84 (−4.48–0.79), 0.171
LRT test	0.027^c^	0.023^c^

a Adjusted for all baseline covariates, other four metals, and gestational age.

b Log-transformed Fe concentration as a continuous variable in the two groups, respectively.

c *p* < 0.05 indicates that Model 2 provides a significantly better fit to the data than Model 1, confirming the significance of the threshold effect.

## 4 Discussion

In this prospective birth cohort study in Northwest China, we found that elevated maternal Ca concentration during early pregnancy was associated with a lower risk of SGA. Additionally, our findings showed that maternal Fe concentration was associated with birth length of male infants in an inverted U-shaped curve. When Fe concentration was less than 7.24 mmol/L (log Fe = 0.86), a one-unit elevation in log-transformed Fe concentration was associated with a 3.23-cm higher birth length in male infants. However, this non-linear relationship was not found in female infants.

Fe deficiency is the predominant micronutrient deficiency worldwide, especially prevalent in low-and middle-income countries (12). Many women of childbearing age already show Fe deficiency prior to pregnancy. In the United States, up to 40% of women have experienced Fe deficiency during early pregnancy (26). 28.1% of pregnant women in the second trimester and 63.7% in the third trimester were diagnosed as having anemia in China (27), the proportion of anemia due to Fe deficiency was estimated to be 85% (28). In this population in Northwest China, 43.1% of mothers had Fe concentrations below the recommended minimum value of 7.6 mmol/L during early pregnancy. The results of a nationwide epidemiological survey carried out in 26 cities and counties revealed a prevalence rate of 49.57% for Fe deficiency during pregnancy (29). The possible explanation for the relatively lower prevalence of Fe deficiency in this study were improvements in living standards and increased health awareness in recent years (30). In general, numerous obstetricians recommend Fe supplementation for pregnant women during the second and third trimesters, as the need for Fe significantly increases in these periods to support the proliferation of maternal red blood cells and fetal development (31). However, they sometimes neglect the possible adverse effects of Fe deficiency in the early stages of pregnancy Table 4.

Our study observed an inverted U-shaped curve between maternal Fe concentration during early pregnancy and birth length. Similar to many other nutrients, Fe presents a U-shaped risk curve in relation to pregnancy outcomes, as Fe overload could lead to a decrease in the absorption of other elements and an increase in the formation of reactive oxygen species, which in turn causes oxidative stress and activates ferroptosis (32). The absence of an association between Fe concentrations in the upper tail and birth length could potentially be explained by the fact that only a small proportion (1.03%) of pregnant women had Fe levels exceeding the recommended range of 9.85 mmol/L in this study. Similar associations between Fe and birth length were demonstrated in several previous studies. A placebo-controlled trial carried out in Southern India demonstrated a notable connection between maternal Fe deficiency and a reduction in birth length (33). In West Java, Indonesia, the consumption of ferrous sulfate by pregnant women per week was associated with an average increase of 1 centimeter in neonatal length (34). The results of animal experiments also indicated that fetuses born to mice on a Fe-deficient diet showed decreased levels of Fe in the fetal brain and shorter crown-rump lengths (35, 36). Moreover, administering lactoferrin to pregnant rats led to improved bone mineral density in their offspring (37).

However, certain studies did not discover any associations between maternal Fe levels and birth length. For example, a birth cohort study on the Tibetan Plateau did not find a significant relationship between Fe concentrations in the third trimester and birth length when urine samples were examined (38). In rural Uganda, there was no significant correlation detected between maternal Fe status (ferritin and body Fe stores) and birth length (39). The inconsistent findings might be ascribed to differences in population, biological specimens, gestational age at testing, and assessment criteria. Moreover, this study discovered that there were varying correlations between maternal Fe and birth length depending on the fetal gender. There was evidence to support the existence of disparities in fetal development vulnerabilities according to biological sex, male infants were more likely to be affected by maternal poor health conditions (40, 41). A birth cohort study conducted in Canada also discovered that the relationships between maternal Fe biomarkers and birth outcomes might be affected by the timing of pregnancy and the sex of the infant (42).

The possible mechanisms responsible for the relationship between maternal Fe status during pregnancy and fetal length growth are as follows. Inadequate supply of Fe could lead to a reduction in the oxygen-carrying capacity of pregnant women, hinder placental development, and impair the transfer of nutrients to the fetus, which ultimately contributes to suboptimal growth of the newborns (43). The primary biological activity of Fe is manifested in its ability to efficiently transfer electrons. It serves as a catalytic cofactor in various biochemical reactions and is crucial for the growth, proliferation, and differentiation of fetal bone cells (44). Maternal Fe deficiency might cause the expression and regulation of fibroblast growth factor-23 to be disrupted, potentially resulting in hypophosphatemia-induced musculoskeletal complications such as rickets in offspring and osteomalacia with fragility fractures in women (45).

Dietary Ca deficiency, which is estimated to affect around 50% of the global population, is a widespread global health issue. The prevalence of Ca deficiency is notably high in low-income and developing countries, with approximately 90% of the 3.5 billion people at risk of insufficient intake of Ca residing in Asia and Africa (46). The correlation between maternal Ca and a decreased risk of SGA observed in this study is in line with the findings of some studies but conflicts with those of others. Maternal ionized Ca lower than 1.31 mmol/L was determined to markedly increase the probability of infants with low birth weight, shorter birth length, and smaller brachial circumference (20). Among low-income and minority pregnant women, it was discovered that maternal Ca metabolic stress was associated with an increased probability of having SGA infants and a significant reduction in birth weight (47). A cross-sectional study also revealed that antenatal Ca supplementation for childbearing-age women in Shaanxi province, China, reduced the risk of having singleton infants with SGA (48). Conversely, results from a Mendelian randomization analysis indicated that maternal Ca during pregnancy does not have a substantial influence on birth weight (49). Furthermore, a study that revealed ethnic differences in the connections between calcium and crucial perinatal outcomes (50) emphasizes the necessity for more in-depth exploration of the relationship between Ca levels and birth outcomes.

To the best of our knowledge, this study is rare in revealing a non-linear inverted U-shaped curve relationship and the threshold effect between maternal Fe levels in early pregnancy and the birth length of male infants in the Chinese population. Additionally, we observed that elevated maternal Ca during early pregnancy was linked to a reduced risk of SGA. Apart from the middle and late stages of pregnancy when anemia is likely to occur and nutrient requirements are increased, the deficiency of Fe and Ca in the first trimester should also receive significant attention in maternal and child health care. Our study also has limitations, the assessment of exposure to minerals was entirely dependent on whole blood samples collected during early pregnancy, which may not offer a comprehensive portrayal of the mineral status of mothers. In addition, the association between Ca concentration and SGA, characterized by an extremely large effect size and wide confidence interval, should be interpreted with caution. This may be due to influential observations in the log-transformed Ca concentration distribution, unmeasured residual confounding, and the inherent instability of estimates given the low SGA prevalence (4.6%). Consequently, our finding requires confirmation in independent cohorts before definitive conclusions can be drawn. Future prospective high-quality multicenter studies, biological marker measurements, and mechanistic research should investigate the impact of mineral nutrition patterns on neonatal growth by pregnancy trimester, comprehensively adjust for confounders, verify the authenticity of these associations, and enhance the development of nutrition intervention strategies.

## 5 Conclusion

In conclusion, elevated maternal Ca during early pregnancy is significantly linked to a lower risk of SGA. Additionally, an inverted U-shaped curve and threshold effects have been noted between maternal Fe concentration and birth length of male infants. This study underscores the necessity for tailored maternal mineral nutrition management strategies and routine monitoring during pregnancy to improve neonatal health.

## Data Availability

The raw data supporting the conclusions of this article will be made available by the authors, without undue reservation.
