# Supplementary material for: Maternal mineral nutrition during early pregnancy and neonatal growth: findings from a China birth cohort study
Source: Front Nutr. 2026 Feb 18;13:1733235. doi: 10.3389/fnut.2026.1733235 (PMC12956538; doi:10.3389/fnut.2026.1733235)
Supplement: Supplementary file 1 [file Data_Sheet_1.PDF]

Supplementary Material

1 Supplementary Figures and Tables

1.1 Supplementary Figures

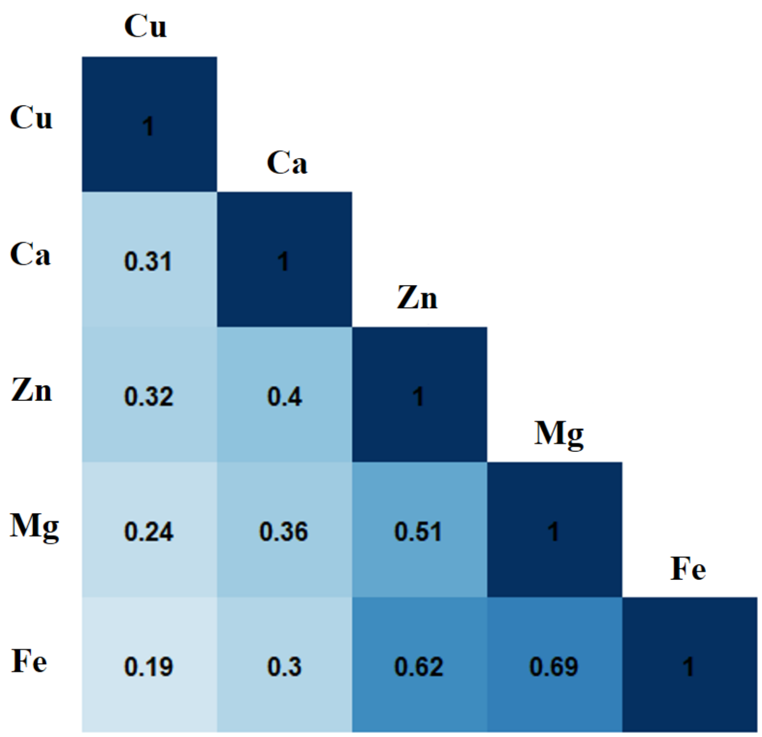

Supplementary Figure 1. Spearman correlation coefficients between minerals.

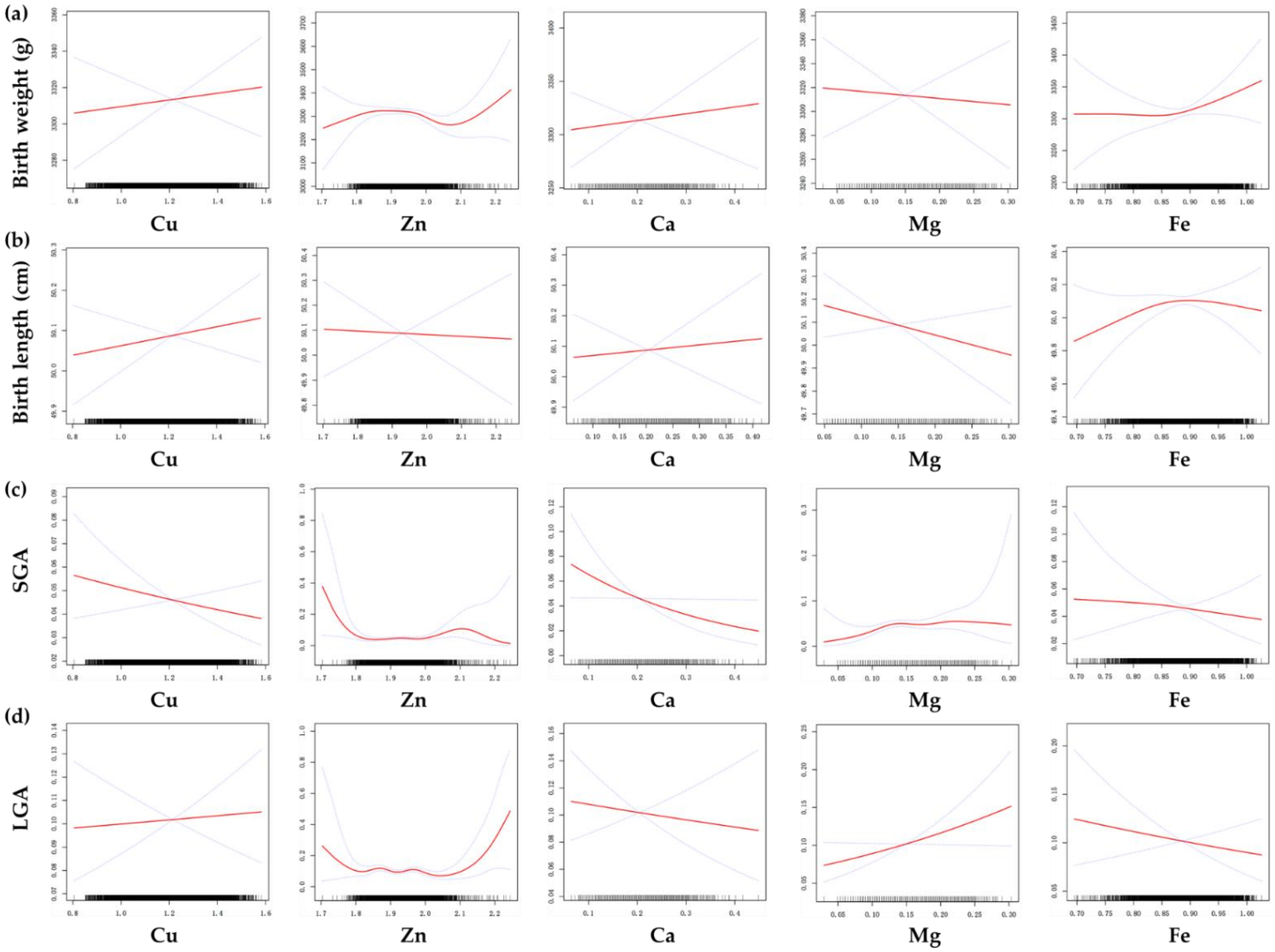

Supplementary Figure 2. Associations of log-transformed maternal mineral nutrition with neonatal birth weight (a), birth length (b), SGA (c), and LGA (d) by smoothing spline.

Note: P-values for nonlinearity: Zn vs. birth weight (P=0.305), Fe vs. birth weight (P=0.419), Fe vs. birth length (P=0.042), Zn vs. SGA (P=0.079), Mg vs. SGA (P=0.096), and Zn vs. LGA (P=0.122).

1.2 Supplementary Tables

Supplementary Table 1 Comparison of baseline characteristics between women included and excluded in the study.

Maternal characteristics	Women included N=5629, n (%)	Women excluded N=21315, n (%)	χ^2	P
Age, years			3.853	0.052
<35	5153 (91.54)	19332 (90.70)		
≥35	476 (8.46)	1983 (9.30)		
Ethnicity			1.547	0.214
Han	5527 (98.19)	20979 (98.42)		
Other	102 (1.81)	336 (1.58)		
Education			5.147	0.076
Senior high school or lower	703 (12.49)	2903 (13.62)		
Junior or regular college	4082 (72.52)	15311 (71.83)		
Graduate or above	844 (14.99)	3101 (14.55)		
Occupation			3.914	0.141
Working regularly	4420 (78.52)	16474 (77.29)		
Workers/peasants/migrant workers	76 (1.35)	310 (1.45)		
Unemployed1	1133 (20.13)	4531 (21.26)		
Household income, CNY			4.622	0.099
Poverty (<50,000)	685 (12.17)	2503 (11.74)		
Medium (50,000~200,000)	3675 (65.29)	13722 (64.38)		
Rich (>200,000)	1269 (22.54)	5090 (23.88)		
First pregnancy			1.195	0.274
No	2547 (45.25)	9471 (44.43)		
Yes	3082 (54.75)	11844 (55.57)		
Mode of conception			1.974	0.373
Spontaneous conception	5254 (93.34)	19887 (93.30)		
Artificial fertilization	34 (0.60)	166 (0.78)		
In vitro fertilization	341 (6.06)	1262 (5.92)		

Supplementary Table 2 Threshold effects of maternal mineral concentrations on neonatal growth outcomes.

Minerals	Birth weight	Birth length	SGA	LGA
	Adjusted β (95% CI) [a], P	Adjusted β (95% CI) [a], P	Adjusted RR (95% CI) [b], P	Adjusted RR (95% CI) [b], P
**Cu**				
Model 1				
One line slope	18.41 (-56.15–92.96), 0.629	0.11 (-0.18–0.41), 0.453	0.60 (0.22–1.55), 0.177	1.11 (0.58–2.24), 0.385
Model 2				
Turning point (K)	1.06	1.43	1.03	1.32
<K [c] slope 1	-202.99 (-524.86–118.87), 0.2165	0.15 (-0.17–0.46), 0.3607	27.11 (0.08–8833.16), 0.264	1.43 (0.62–3.29), 0.396
≥K [c] slope 2	59.42 (-35.04–153.87), 0.2176	-0.92 (-4.15–2.32), 0.5783	0.38 (0.12–1.25), 0.111	0.33 (0.03–3.84), 0.376
LRT test [d]	0.165	0.529	0.168	0.308
**Zn**				
Model 1				
One line slope	-140.29 (-347.67, 67.08), 0.185	-0.12 (-0.95–0.71), 0.781	3.72 (0.17–36.40), 0.212	0.41 (0.04, 2.89), 0.216
Model 2				
Turning point (K)	1.86	1.86	1.95	1.98
<K [c] slope 1	542.05 (-420.18–1504.29), 0.270	1.40 (-2.50–5.30), 0.482	0.22 (0.00–66.70), 0.607	1.99 (0.07–55.67), 0.6860
≥K [c] slope 2	-203.35 (-428.16–21.45), 0.076	-0.26 (-1.16–0.65), 0.578	–	–
LRT test [d]	0.154	0.435	0.072	0.257
**Ca**				
Model 1				
One line slope	51.64 (-199.81–303.09), 0.687	0.17 (-0.84–1.18), 0.744	0.03 (0.01–0.80), 0.034	0.49 (0.04–4.32), 0.274
Model 2				
Turning point (K)	0.16	0.16	0.15	0.17
<K [c] slope 1	-829.70 (-2050.48–391.09), 0.183	-2.55 (-7.49–2.38), 0.311	–	0.01 (0.00–12.34), 0.193
≥K [c] slope 2	135.44 (-140.45–411.33), 0.336	0.42 (-0.68–1.53), 0.454	0.01 (0.00–0.41), 0.015	1.19 (0.09–16.15), 0.894
LRT test [d]	0.147	0.269	0.088	0.253

**Mg**				
Model 1				
One line slope	-44.56 (-378.74–289.61), 0.794	-1.13 (-2.47–0.20), 0.096	24.11 (0.85–117.63), 0.054	9.20 (0.78–27.10), 0.070
Model 2				
Turning point (K)	0.15	0.23	0.12	0.15
<K [c] slope 1	-364.23 (-964.16–235.69), 0.234	-1.52 (-2.93–-0.10), 0.035	–	4.43 (0.02–907.00), 0.584
≥K [c] slope 2	195.89 (-306.23–698.02), 0.445	7.90 (-3.09–18.90), 0.159	3.14 (0.02–515.78), 0.660	21.14 (0.30–1504.29), 0.161
LRT test [d]	0.208	0.104	0.052	0.688
**Fe**				
Model 1				
One line slope	159.04 (-144.73–462.81), 0.305	0.53 (-0.68–1.75), 0.390	0.18 (0.01–8.27), 0.213	0.32 (0.02–4.38), 0.255
Model 2				
Turning point (K)	0.85	0.86	0.84	0.96
<K [c] slope 1	-183.62 (-779.42–412.18), 0.546	2.42 (0.35–4.49), 0.022	–	0.40 (0.03–5.91), 0.506
≥K [c] slope 2	354.07 (-67.08–775.23), 0.099	-0.93 (-2.71–0.85), 0.307	0.03 (0.00–4.98), 0.174	–
LRT test [d]	0.189	**0.027**	0.255	0.660

[a] Adjusted for all baseline covariates, other four metals, and gestational age.
[b] Adjusted for all baseline covariates and other four metals.
[c] Log-transformed mineral concentrations as a continuous variable in the two groups respectively.
[d] $P<0.05$ indicates that Model 2 provides a significantly better fit to the data than Model 1, confirming the significance of the threshold effect.
Note: Some estimates are unstable due to limited sample size in certain strata, as evidenced by wide confidence intervals or non-estimable results marked as '–'.

Supplementary Table 3 Threshold effects of maternal Fe concentration on birth length in female infants.

Birth length	Adjusted β (95% CI) [a], P
Model 1	
One line slope	0.66 (-0.95–2.27), 0.442
Model 2	
Turning point (K)	0.85
<0.85 [b] slope 1	1.67 (-1.53–4.87), 0.306
≥0.85 [b] slope 2	0.07 (-2.20–2.35), 0.952
LRT test	0.471 [c]

[a] Adjusted for all baseline covariates, other four metals, and gestational age.
[b] Log-transformed Fe concentration as a continuous variable in the two groups respectively.
[c] P<0.05 indicates that Model 2 provides a significantly better fit to the data than Model 1, confirming the significance of the threshold effect.
